# The development of a digital story-retell elicitation and analysis tool through citizen science data collection, software development and machine learning

**DOI:** 10.3389/fpsyg.2023.989499

**Published:** 2023-04-20

**Authors:** Rebecca Bright, Elaine Ashton, Cristina Mckean, Yvonne Wren

**Affiliations:** ^1^Therapy Box, London, United Kingdom; ^2^School of Education, Communication and Language Sciences, Newcastle University, Newcastle upon Tyne, United Kingdom; ^3^North Bristol NHS Trust, Bristol, United Kingdom; ^4^Bristol Dental School, University of Bristol, Bristol, United Kingdom; ^5^Cardiff School of Sport and Health Sciences, Cardiff Metropolitan University, Cardiff, United Kingdom

**Keywords:** story retell, citizen science, language sample, machine learning, speech pathology, story grammar

## Abstract

**Background:**

In order to leverage the potential benefits of technology to speech and language therapy language assessment processes, large samples of naturalistic language data must be collected and analysed. These samples enable the development and testing of novel software applications with data relevant to their intended clinical application. However, the collection and analysis of such data can be costly and time-consuming. This paper describes the development of a novel application designed to elicit and analyse young children’s story retell narratives to provide metrics regarding the child’s use of grammatical structures (micro-structure) and story grammar (macro-structure elements). Key aspects for development were (1) methods to collect story retells, ensure accurate transcription and segmentation of utterances; (2) testing the reliability of the application to analyse micro-structure elements in children’s story retells and (3) development of an algorithm to analyse narrative macro-structure elements.

**Methods:**

A co-design process was used to design an app which would be used to gather story retell samples from children using mobile technology. A citizen science approach using mainstream marketing *via* online channels, the media and billboard ads was used to encourage participation from children across the United Kingdom. A stratified sampling framework was used to ensure a representative sample was obtained across age, gender and five bands of socio-economic disadvantage using partial postcodes and the relevant indices of deprivation. Trained Research Associates (RA) completed transcription and micro and macro-structure analysis of the language samples. Methods to improve transcriptions produced by automated speech recognition were developed to enable reliable analysis. RA micro-structure analyses were compared to those generated by the digital application to test its reliability using intra-class correlation (ICC). RA macro-structure analyses were used to train an algorithm to produce macro-structure metrics. Finally, results from the macro-structure algorithm were compared against a subset of RA macro-structure analyses not used in training to test its reliability using ICC.

**Results:**

A total of 4,517 profiles were made in the app used in data collection and from these participants a final set of 599 were drawn which fulfilled the stratified sampling criteria. The story retells ranged from 35.66 s to 251.4 s in length and had word counts ranging from 37 to 496, with a mean of 148.29 words. ICC between the RA and application micro-structure analyses ranged from 0.213 to 1.0 with 41 out of a total of 44 comparisons reaching ‘good’ (0.70–0.90) or ‘excellent’ (>0.90) levels of reliability. ICC between the RA and application macro-structure features were completed for 85 samples not used in training the algorithm. ICC ranged from 0.5577 to 0.939 with 5 out of 7 metrics being ‘good’ or better.

**Conclusion:**

Work to date has demonstrated the potential of semi-automated transcription and linguistic analyses to provide reliable, detailed and informative narrative language analysis for young children and for the use of citizen science based approaches using mobile technologies to collect representative and informative research data. Clinical evaluation of this new app is ongoing, so we do not yet have data documenting its developmental or clinical sensitivity and specificity.

## Introduction

The study of children’s language acquisition has a long history. This fundamental developmental achievement has been scrutinised by scholars from many different disciplines, including psychology, linguistics, education and speech and language pathology. A key method to examine children’s language learning, which has yielded crucial insights since the inception of audio-recording technology, is to record a sample of a child’s interaction, transcribe the language heard, and analyse the linguistic structures used by the child (Bernstein Ratner and MacWhinney, 2019). Although not without challenges, recording and analysing a language sample has long been recognised as an ecologically valid measure of a child’s language abilities in a functional context (Miller, 1996). In addition to research contexts, the analysis of language samples yields important insights for speech and language therapists/pathologists who work with individuals with language disorders.

### Language samples and their place in clinical practice

Transcribing and analysing samples of a child’s spoken language supports clinicians in evaluating performance with reference to typical development, undertaking goal-setting, and measuring progress. It is seen by some as the “gold standard” for analysing a child’s language skills with advantages over standardised testing procedures, including a more naturalistic assessment of a child’s ability and potentially providing less culturally biased measures of a child’s development (Heilmann et al., 2010). It is also possible to repeat a language sample assessment more frequently than a standardised test without any threat to the validity or reliability of the procedure, and so enable evaluation of progress over time (Wilder and Redmond, 2022). Samples of narratives and story re-tells are particularly informative contexts for linguistic analysis in terms of their ability to distinguish between diagnostic subgroups (Botting, 2002) due to the high processing demands they place on the speaker to uncover impairments (Wagner et al., 2000). Importantly, they are also a very sensitive predictor of prognosis in both language and literacy outcomes in children with early language difficulties (Bishop and Edmundson, 1987; Botting, 2002; Miller et al., 2006). Analysis of narratives and story retells can focus on micro-structure elements, such as grammatical morphology, syntax and vocabulary and macro-structure features, related to the overarching organisation and coherence of the story, sometimes referred to as ‘story grammar’ (Westerveld and Gillon, 2010; Gillam et al., 2017). The former is highly informative to the clinician concerning the presence and nature of semantic and morpho-syntactic deficits and their impacts on functional communication; the latter brings insights related to discourse and pragmatic abilities.

Recent changes to diagnostic criteria for Developmental Language Disorder (DLD) bring a renewed focus on methods to evaluate a child’s ability to use language *functionally* in context. A DLD diagnosis is not determined by cut-points on standardised tests but rather by a language problem that ‘causes functional impairment in everyday life’, (Bishop et al., 2017 p. 1068). Few rigorous and reliable assessment methods exist for identifying such functional impairments. Language sampling and analysis offer such a method; however, many barriers prevent its widespread use in clinical practice.

### Barriers to the use of language sampling in practice

Despite numerous calls for clinical practice to change, so that language sampling, transcription and detailed analysis become standard practice, barriers of time, skills, knowledge and confidence levels continue to prevent this (Kemp and Klee, 1997; Westerveld, 2014; Pavelko et al., 2016; Pezold et al., 2020; Klatte et al., 2022). Training alone has been insufficient in leading to increased use of language sample analysis, despite clinicians having an awareness of the benefits (Klatte et al., 2022). Therefore, barriers other than skills and knowledge also need to be addressed.

### The use of technology to support the use of language sample analysis in clinical practice

Computer-based language sample analysis is a way to gather qualitative information about a child’s language that complements other assessment processes (Pezold et al., 2020; Klatte et al., 2022). Furthermore, the use of technology to semi-automate processes of transcription and analysis has the potential to ameliorate barriers of time and perhaps to scaffold and support clinicians who are less confident in linguistic analysis. However, despite the presence of existing software and training programs, clinicians report that the hurdles described above persist (Klatte et al., 2022). Hence, currently available tools are not yet suited to clinical practice in terms of ease of use and time demands. Klatte et al. (2022) suggested that language sampling software developed in codesign with clinicians and shorter narrative-based sampling could overcome some of the identified obstacles.

Challenges also exist in developing automated language analysis technology which can provide clinicians with the relevant analysis of micro and macro structures required to inform diagnosis and intervention. Micro-structure elements vary in the degree of challenge they present to automated analysis depending on the ambiguity and potential for miscategorisation. Identifying a determiner such as ‘the’ is relatively easy, a bound morpheme such as -ed is more complex, and a copula, whose identification rests on the surrounding context, is substantially more challenging. Macro-structure, or ‘story grammar’, is evaluated through the identification of the presence of the description by the speaker of factors such as the story setting, the initiating event, and the characters’ internal response, together with a rating of the success or sophistication of the language used to describe those elements (Westerveld and Gillon, 2010). Potential automation to assist in this process requires the software to recognise the many different ways a speaker might encode an internal response or a setting and ascribe a relatively subjective rating to them.

### The use of citizen science to support the development of an automated language analysis tool

Citizen science approaches involve members of the public as collaborators in scientific research, such as in formulating research questions, data collection or analysis of findings (Bonney et al., 2009). The relatively low cost of mobile app based data collection, high-quality audio recording, and attractive ‘gamified’ data elicitation procedures (Gillan and Rutledge, 2021) bring unprecedented opportunities to gather large-scale naturalistic language data. Furthermore, targeted marketing campaigns can enable geographical and socio-economic reach that may otherwise be difficult or costly. In this way, citizen science approaches enable the development and testing of novel software applications with large-scale data relevant to their intended clinical and research application. While citizen science approaches offer an attractive means of gathering data at low cost and quickly from a broad group of participants, limitations include variability in data and potential differences in how similar data would be collected in person by researchers. Here we examine the potential of such approaches to be used to develop a language sampling and analysis tool.

### The current study

A product or tool that supports story retell elicitation, automated speech recognition, transcription improvement and language analysis is yet to be realised (Scott et al., 2022). This study takes the first steps in developing such a tool for language sample elicitation, collecting a large representative sample of young children’s naturalistic language and developing and testing the app’s ability to accurately analyse key aspects of the child’s linguistic development.

The Language Explorer data collection app aimed to elicit a language sample *via* a story retell task and provide users with software-based tools to support transcription and analysis of micro and macro-structure elements of the samples. Supported by funding from an NIHR i4i Product Development Award, software was co-designed with children and clinicians. To develop a reliable and valid tool, we needed to collect large-scale data representing the likely range of ages and language abilities we would see in the clinical context for which the tool was intended. This would allow micro-structure analytical methods to be refined and a macro-structure analysis algorithm to be trained. To ensure Language Explorer could be used reliably in practice, we also needed to ensure it was acceptable to families. In 2020 we embarked on a Citizen Science study with two stages: (1) to collect a representative sample of United Kingdom children’s story retelling using the Language Explorer app and (2) to complete the development of the language transcription and analysis tool.

We aimed to address the following research questions:

- Is it possible to gather a representative stratified sample of story retell recordings of children aged 4–7 years across the United Kingdom using Citizen Science methods?
- How acceptable is the Language Explorer App to families participating in the citizen science project?
- Is the quality of the recordings sufficient for reliable transcription and analysis?
- What level of reliability in automated micro-structure analyses can be achieved?
- Is it possible to develop a software platform that can provide reliable macro-structure analyses? If so, what level of reliability can be achieved?

The following presents the methods and results for each stage of the citizen science project, including data collection and analysis. The clinical evaluation of the tool will be reported in later publications.

## Methods

**Figure 1:**
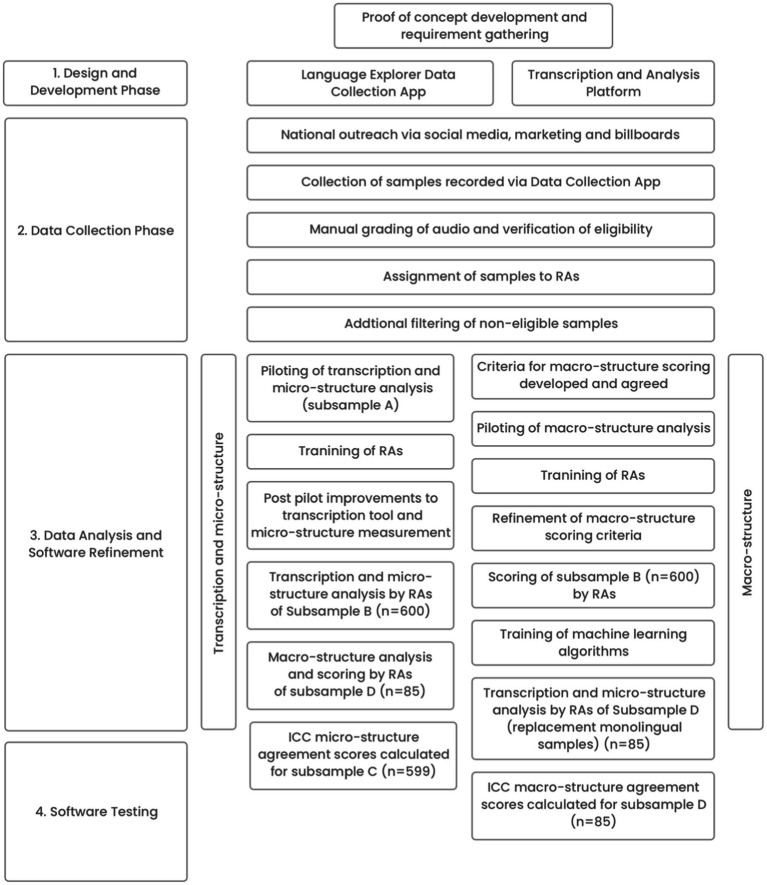
Project phases.

The study had four phases (1) design and development; (2) language sample data collection; (3) data analysis and software refinement and (4) software testing. Phases 3 and 4 involved different methods for the macro and micro-structure elements. The phases, their linkage and their sub-components are represented in Figure 1.

### Design and development

#### Codesign of the language explorer data collection app

Using principles of user-centred design and co-design, the design team worked with clinicians, parents and children of primary school age to develop the content for the story retelling stimulus. The semi-animated story of a boy on a treasure quest underwent usability testing with children. Using a standard usability testing approach (Gomoll, 1990; Norman and Kirakowski, 2018) children, clinicians and parents at two Hackney schools were provided with the app on iPads, given an overview of the app’s purpose, provided with instructions to use the app from start to finish and observed using it. Verbal feedback and observations relating to engagement, accessibility and ease of use were collected. Based on the usability testing, instructions were refined, button sizes adjusted and user experience design elements were added to make progressing through the app more intuitive. The story script was developed following advice from researchers with specialist knowledge of syntactic structures likely to be challenging to children with language disorders. A survey to elicit parent feedback during the citizen science phase was also included (see Appendix 1).

#### Codesign of the transcription and micro-structure analysis platform

Proof of concept work examined the potential for using speech recognition for child language sampling and analysis was completed. ‘Requirement gathering’ was completed to determine what a project needs to achieve and what needs to be created to make that happen. Feedback was elicited from clinicians on early-stage prototypes with iterative improvements in the design of the transcription improvement tool (to manually improve the accuracy of transcription provided by the Automatic Speech Recognition software) and micro-structure analysis software between workshops.

### Language sample data collection

A United Kingdom wide campaign was undertaken to call for participants to crowdsource samples using the Language Explorer app. Ethical approval was provided by Bristol University (reference 97,304). Outreach *via* social media, press and paid targeted marketing was conducted. In addition, the use of location-targeted billboards was designed to attract attention to the study. The outdoor media was placed to take advantage of the expected traffic of parents with children within the target age range, including within a short range of schools and transport hubs. Campaign messaging encouraged participants to contribute to the study to help children with language disorders in the future.

The app was downloadable from the AppStore and PlayStore. Consent for the data to be used for research was sought *via* the app. Recordings were transferred to a designated data management platform meeting GDPR requirements. Families could complete the task offline with data only uploaded when they were next online to reduce reliance on connectivity. In addition to the audio recordings, demographic data were entered in the app by the end-user, presumed to be the parent/carer key to enable stratification of the sample and consideration of exclusion and inclusion criteria for data analysis. These were the child’s age, country, partial postcode, whether the child had a diagnosed communication difficulty or disorder or other disability and the languages spoken in the home (see Appendix 1). A proportionate stratified sampling approach was used to gather participants to use in the piloting and training of the software such that it would cover children across the United Kingdom equally distributed by sex, five age bands (4:0–4:5; 4:6–4:11; 5:0–5:11; 6:0–6:11; and 7:0–7:11) and quintiles of socio-economic disadvantage (see Table 1 for planned sample). The latter was defined using partial postcodes. Partial postcodes were used to enable stratification whilst remaining at a level of granularity unlikely to raise concerns amongst participants regarding confidentiality and data protection. The 2019 Indices of Multiple Deprivation (IMD) for each United Kingdom nation were consulted (McLennan et al., 2019). National quintiles for each partial postcode area were created by averaging the IMD ranking of all postcodes represented within the partial postcode area. Participants’ partial postcodes were then mapped to these quintiles.

**Table 1:** Target stratified sampling frame.

Age	4–4:05	4:06–4:11	5:0–5:05	5:06–5:11	6–6:11	7–8
	*N* = 100	*N* = 100	*N* = 100	*N* = 100	*N* = 100	*N* = 100
IMD Q1	20	20	20	20	20	20
IMD Q2	20	20	20	20	20	20
IMD Q3	20	20	33	20	20	20
IMD Q4	20	20	20	20	20	20
IMD Q5	20	20	20	20	20	20
	M (50)	M (50)	M (50)	M (50)	M (50)	M (50)
	F (50)	F (50)	F (50)	F (50)	F (50)	F (50)

IMD, indices of multiple deprivation (Office for National Statistics, 2019); *Q*, quintile where 1 is the least deprived and 5 is the most deprived.

Samples from outside of the United Kingdom or where children spoke a language other than English were excluded from further consideration at this phase, as were children with an identified disability or communication disorder. The focus on monolingual typically developing children at this stage was to enable valid comparison of these data to the planned clinical evaluation population. Recruitment continued until all strata contained the target numbers with the desired characteristics. For example, 20 children aged 4–4:05 in each IMD quintile made up of 10 girls and 10 boys (see Table 1).

#### Sample achieved

**Figure 2:**
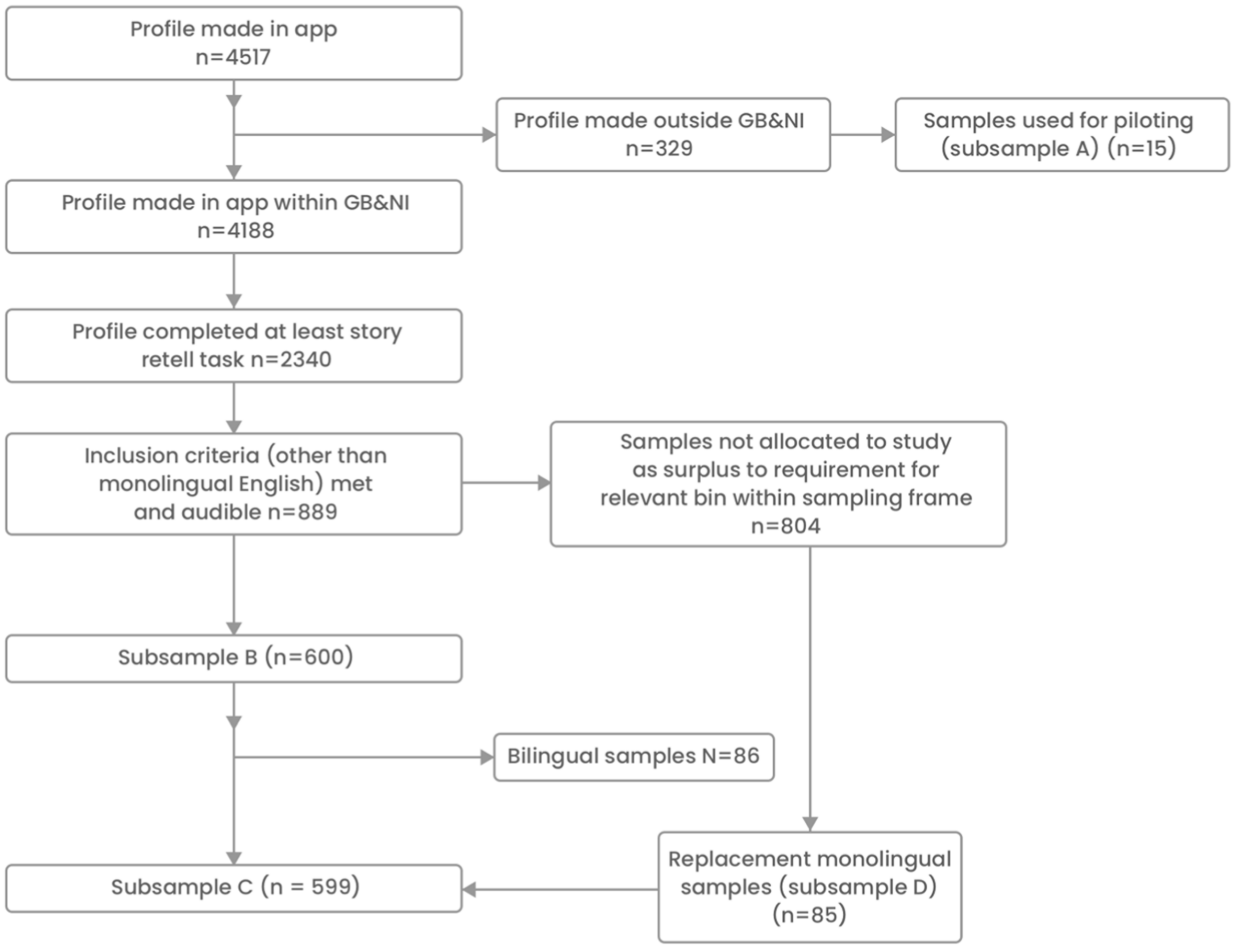
Participant flow chart.

In achieving the stratified sample, 4,517 profiles were made in the Language Explorer data collection app (Figure 2). Following the exclusion of 329 profiles registered as being from outside Great Britain or Northern Ireland, a total of 4,188 profiles remained. Of the 4,188 profiles, 2,340 had completed the story retelling, sentence comprehension and repetition tasks. The initial exclusion of participants who were not English first language participants and/or listed as having a communication or other disability left 1,451 samples. A further 312 participants that would have otherwise been eligible were excluded as they were not graded as audible when samples were screened manually. Samples graded as audible totalled 889.

The obtained recordings were used for differing purposes with different subsamples of children drawn from the pool of 889 as appropriate to the purpose of the work.

Subsample A: Piloting and micro-structure analysis refinement – a sample of 15 English-speaking children from outside of the United Kingdom surplus to requirement for the stratified sample but of sufficient quality for piloting and for RA training.

Subsample B: Development of the macro-structure algorithm – 600 children either monolingual or bilingual with English as a first language who met our stratification strategy with respect to gender, IMD and age. This was made up of 86 bilingual children and 514 monolingual children. It was proving difficult to identify monolingual children fitting precisely into the required stratified sampling frame with respect to age, gender and IMD. Including English first language bilingual children allowed the development of the algorithm to proceed whilst monolingual replacement samples were sought.

Subsample C: Testing of the micro-structure analysis – 599 monolingual children who met our stratification strategy with respect to age, IMD and gender. A monolingual only sample was required for the testing phase to better align with the participants expected to be recruited for the later clinical evaluation. Therefore this sample is made up of the 514 monolingual children in subsample B plus new monolingual children with the same age, gender, IMD characteristics as the 86 bilingual children dropped from subsample B. We identified 85 appropriate monolingual children giving a total of 599 in this subsample.

Subsample D: Testing of the macro-structure analysis – 85 monolingual children not used in the development of the macro-structure analysis algorithm also used in subsample C.

### Data analysis and software refinement – Micro-structure

Anonymous recordings were received by the research team in the data management platform. Data were then analysed by a tea of four research associates: three junior research assistants (RAs) who were all clinically qualified SLTs and one lead RA, also a qualified SLT and with an additional qualification in linguistics, all based at Newcastle University. Following checking of audio, samples in each stratum were allocated to one of the three RAs, each completing transcription and analysis. RA1 completed transcribed and analysed 36% of the samples, RA2 44% ad RA 3 19%. The lead RA carried out reliability checking of 10% of samples. RAs were ‘blind’ to the age, gender and other demographic data of the sample. A further quality assurance process took place at this stage, where the RAs judged several samples not to be suitable for language analysis. Samples were excluded where the story’s content was too short, the narrative was incomplete, where it was judged there was too much secondary speaker input, which limited the child’s performance, for example, the adult retold the story and the child repeated what the adult had said, or where a non-English language was spoken. These samples were flagged and removed, and replacement samples meeting the same demographic criteria necessary for the stratification sample were allocated. The RAs discussed with the lead RA if they had questions about the inclusion of a sample.

The goal was to compare the Language Explorer analysis of micro-structure components to the Systematic Analysis of Language Transcripts (SALT) software (Miller et al., 2019). SALT is the most widely used language analysis software designed for clinical use and has high levels of validity and reliability (Tucci et al., 2022). The SALT software requires the researcher or clinician to transcribe and annotate the transcript following particular conventions and then automatically calculates certain micro-structure features in language samples, e.g., the number of adjectives and prepositions. It also allows the user to manually mark other morphological markers that it can then calculate automatically, e.g., plural -s, past tense -ed, and personalised tags such as auxiliary and copula verbs. The RAs needed to be both reliable and consistent in their transcription and utterance segmentation and in the conventions required by the SALT software for accurate analysis. These consist primarily of additional ‘tags’ required to identify key micro-structures in the sample. The three RAs were trained using subsample A - pilot dataset of 15 samples which were not included in any further analysis. The aim was to achieve greater than 85% inter-rater agreement in manual transcription and SALT language analysis before moving on to the samples to be used in the later phases of the method. The lead RA compared each of the RAs’ transcription and analyses of the pilot data with her own, and they reached a level of 97% agreement for the manual transcription and 96% for the micro-structure language analysis completed in SALT, indicating reliable use of transcription conventions across the team.

Using the same subsample A (*N* = 15), the lead RA compared the transcripts from the manually completed pilot samples and the micro-structure analysis from SALT with the samples completed using the Language Explorer clinical software tools. This involved a comparison of transcription using the transcription improvement tool and reviewing the counts of the ‘parts of speech’. Feedback was provided to the software engineers, and improvements were made to the software. Automated measures that did not reach the 85% level of agreement between manual and automated results were scrutinised, and the potential sources of error were discussed to retrain the automated microstructural analysis using the software. The software engineers and the lead RA checked each problematic metric in the 15 pilot samples. The software was modified as a result, including clarifying rules in the software for identifying metrics and providing examples.

### Data analysis and software development: Macro-structure

Unlike the micro-structure analysis, the macro-structure analysis required ongoing refinement using subsample A and subsample B. The macro-structure metrics focussed on seven macro-structure ‘story grammar’ elements (setting, initiating event, internal response, plan, attempt, consequence and character) based on Stein and Glenn (1979). A matrix of definitions and examples was prepared using a scoring system of 0–3 for each element, with 0 being unobserved and 3 being the score allocated for a full demonstration of that macro-structure element. This was used to build and train the algorithms.

Before training the algorithms, high levels of agreement between the RAs were necessary to ensure high-quality data. Substantial training and refinement of the scoring rubric were required to reach the necessary levels of agreement between the RAs. Initially, the levels of agreement of the story grammar scoring between the lead RA and the three RAs were low (ICC of RA1 0.455, RA2 0.562, RA3 0.587). The lead RA therefore refined the macro-structure descriptors and scoring examples using specific examples from the pilot dataset stories, as well as applying learning from other studies (Beswick, 2008; Gillam et al., 2017; Gillam and Gillam, 2018; Jones et al., 2019; Diehm et al., 2020). Due to the more subjective nature of macro-structure scoring and hence challenges in establishing reliability (Calder et al., 2018), a threshold was set for inter-rater agreement in the training phase of 75% for each story grammar component and 85% agreement for the story grammar total score Once this was achieved, the RAs could move on to scoring subsample B (*N* = 600) to train the algorithm.

The training of the RAs used real examples from the pilot to further support learning. It worked in short intervals using sets of three samples from subsample A (*N* = 15) before checking in on agreement and discussing sources of disagreement. A final test set of five additional pilot samples was used following this revised training. The two RAs achieved above the necessary agreement scores with the lead RA (0.885 and 0.940 for the macro-structure (story grammar) elements and 0.938 and 0.938 for the total macro-structure score), noting that one RA left the project at this point on maternity leave. The RAs then scored subsample B (*N* = 600) for story grammar, with the lead RA providing reliability checking of 10% of the samples. The agreement scores for this reliability checking were 0.907 and 0.869 for each of the two RAs for story grammar components and 0.956 and 0.925 for the total story grammar score (Appendix 5). Following completion of the manual scoring, the revised descriptors and scoring data for each macro-structure element and the examples were used to train the algorithms.

#### Software testing: Micro-structure

The RAs completed orthographic transcription and manual analysis of the samples received in the stratified subsample B (*N* = 600). Further reliability checking was carried out with 10% of the transcriptions and SALT language analyses for each RA compared with the lead RA using an identical method to that used for piloting (60 samples in total). The levels of reliability achieved were 93% for the transcription and 98% for the language analyses (Appendix 5), confirming the maintenance of the high levels of reliability achieved during training. Subsample C (*N* = 599) using only monolingual participant samples was used for the analysis of the reliability of the Language Explorer tool for micro-structure features. Intra-class correlations (ICCs) were calculated by comparing each of the parts of speech metrics calculated by the Language Explorer tool and those calculated for comparison *via* manual transcription and SALT analysis.

#### Software testing: Macro-structure

Given the need to use the whole of subsample B (*N* = 600) in the training of the algorithms, testing the performance of the macro-structure analysis module in the clinical tools software was limited to using subsample D (*N* = 85 monolingual replacement samples) that were not used in training. Intra-class coefficient (ICC) scores were calculated for each macro-structure element and the total score for the seven elements.

## Results

Results are presented in turn for phases (1) design and development; (2) language sample data collection, and (3) software testing.

### Design and development

**Figure 3:**
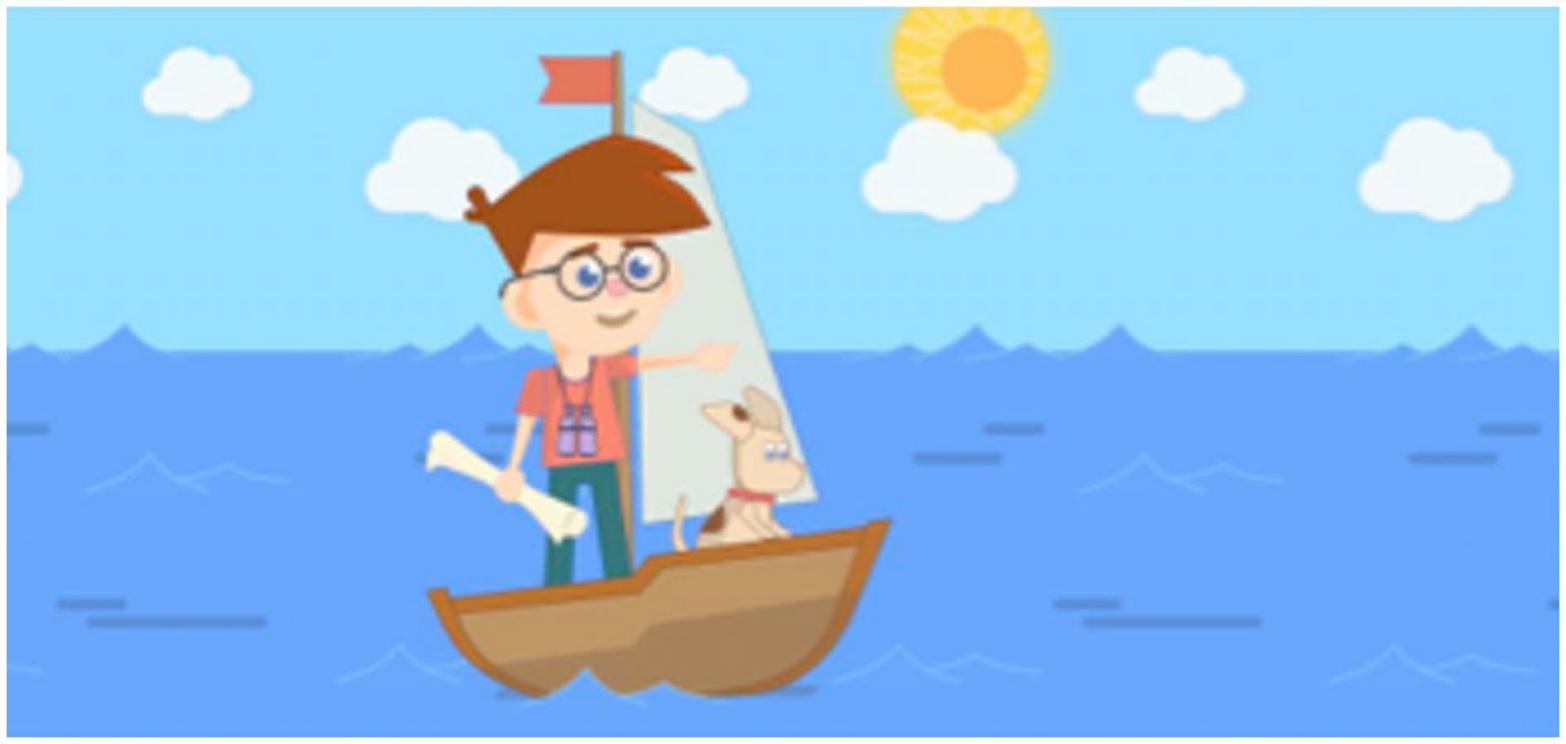
Screen from the language explorer story in the app.

The co-design work with children, parents, researchers and clinicians informed the development of the final Language Explorer Mobile application. This app presents semi-animated story of a boy on a treasure quest and following the story the child is asked to retell the story with pictorial support. This story retell is recorded using inbuild recording technology in the phone or tablet being used. The resulting story was designed to elicit a linguistically rich sample with maximum efficiency. Participants’ parents using the app in the citizen science phase were surveyed and asked about their experience. A total of 426 parents completed the survey. Most parents reported that the app was easy to use (95%) and that their children enjoyed using it (91%) (Figure 3).

The workshops with clinicians provided insights into the desire for technology to make language sampling quicker and easier and the need to collect samples on contemporary mobile technologies. The preference was for any such technology not to be a ‘black-box’ system that produces a ‘score’ without a transparent method but for clinicians to review the analysis and understand the processes and metrics. That is for the app to provide familiar and readily interpretable micro- and macro-structure metrics.

**Figure 4:**
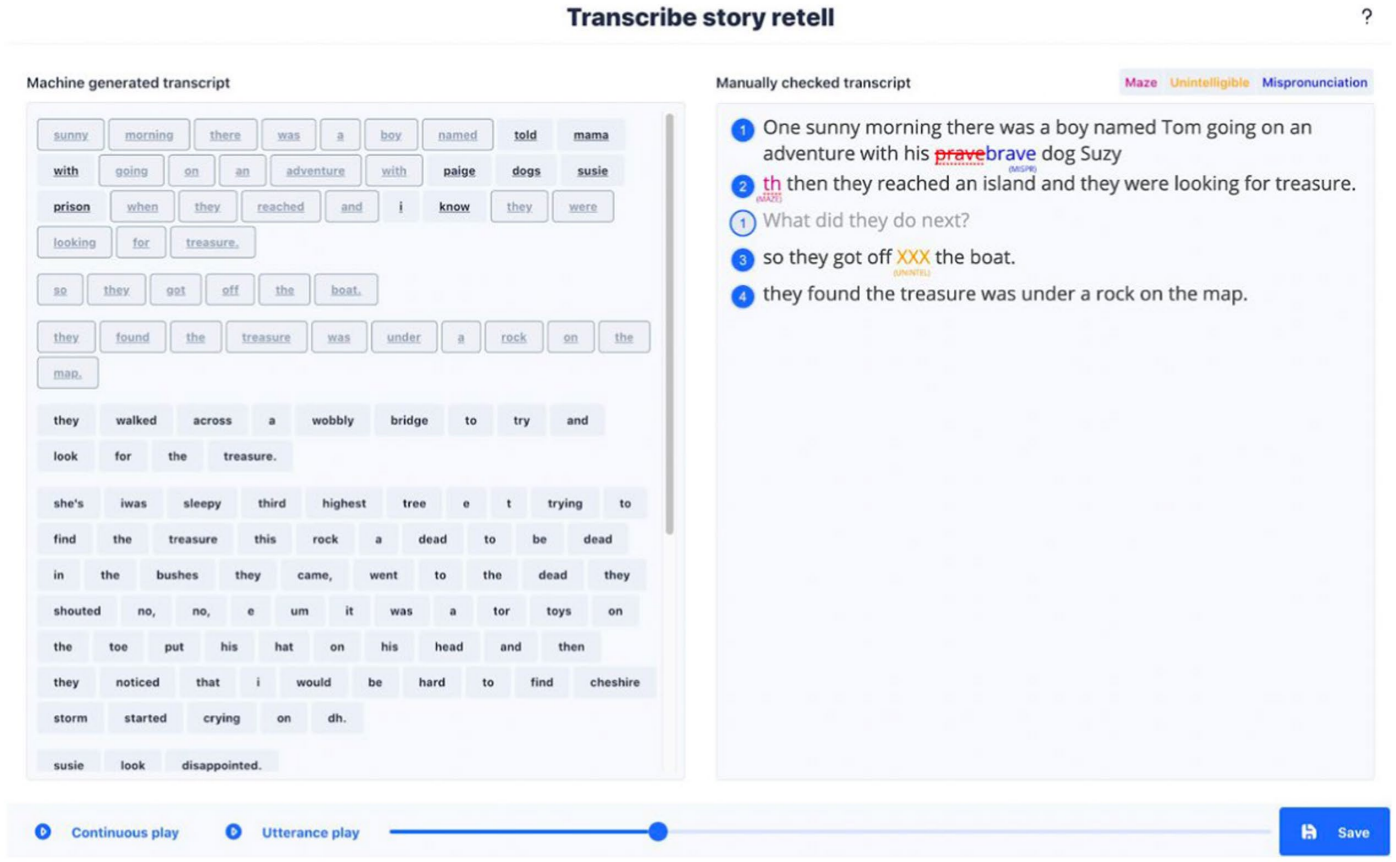
Screen from the transcription improvement tool.

With regards to transcription processes the following features were developed as a result of the co-design and consultation. Upon receiving an audio recording of the story retell from the app, an automated speech recognition (ASR) based transcript is created and presented to the clinician user. Given the current industry accuracy for ASR for child speech (Yeung and Alwan, 2018; Hair et al., 2019), there remains a need to correct and improve the transcriptions. A ‘transcription improvement tool’ was designed and built to allow clinicians to use the ASR transcription and select words or utterances transcribed accurately by the ASR while making any corrections. The clinician at this stage also follows specific conventions to segment utterances, identify secondary speaker utterances and annotate occurrences of mazes, mispronunciations or unintelligible speech. These are necessary to ensure the analysis software can produce accurate micro-structure metrics consistently across speakers and across users checking the transcriptions. After confirming an accurate transcript, clinicians are presented with the same transcript. It is colour-coded by parts of speech with the marking of morpheme boundaries, allowing additional parts of speech to be tagged. There is the possibility at this stage of checking and modification by the clinician (Figure 4).

### Data collection

The geographical distribution across United Kingdom regions of Subsample C used for testing the reliability of the micro-structure analysis was highly similar to that of the United Kingdom population across those regions (Table 2).

**Table 2:** Participant geographical location based on partial postcode compared with population distribution.

United Kingdom region	Participants	Language explorer sample representation	Population distribution	Difference between proportion in sample obtained and United Kingdom population
East Midlands	53	8.8%	7.23%	1.6%
East of England	43	7.2%	9.25%	−2.1%
London	62	10.4%	12.97%	−2.6%
North East	42	7.0%	4.12%	2.9%
North West	78	13.0%	11.14%	1.9%
Northern Ireland	8	1.3%	2.83%	−1.5%
Scotland	30	5.0%	8.38%	−3.4%
South East	67	11.2%	13.63%	−2.4%
South West	82	13.7%	8.37%	5.3%
Wales	20	3.3%	4.82%	−1.5%
West Midlands	57	9.5%	8.91%	0.6%
Yorkshire and The Humber	57	9.5%	8.36%	1.2%
Total	599			

Characteristics of subsample C (*N* = 599) used to test the micro-structure analysis are described in Table 3 Speech duration ranged from 35.66 s to 251.4 s. Table 3 summarises the duration of the samples, their length in total number of words and the number of secondary speaker utterances.

**Table 3:** Duration of speech, length in number of words and number of secondary speaker turns by age bands in the “Final 599” sample.

Age band	Length in speech duration in seconds M (SD)	Length in total number of words M (SD)	Number of Secondary speaker utterances M (SD)
	*N* = 588	*N* = 599	*N* = 599
4:0–4:5	109.48 (37.1)	136.18 (48.9)	11.36 (15.1)
4:6–4:11	106.13 (35.7)	132.58 (45.6)	8.75 (10.4)
5:0–5:5	108.62 (33.7)	145.71 (43.9)	7.26 (10.4)
5:6–5:11	108.53 (34.3)	146.19 (42.9)	4.54 (7.1)
6:0–6:11	109.38 (34.2)	162.67 (54.4)	2.81 (4.3)
7:0–7:11	107.47 (32.1)	167.54 (40.8)	2.42 (4.2)

### Software testing: Micro-structure

The following presents the ICC scores for each micro-structure analysis metric between the RAs and the Language Explorer output computed for subsample B (*N* = 599) (Table 4). Note the use of full transcript and analysis set scoring differs in that the analysis set scores were calculated after the removal of incomplete utterances and utterances containing unintelligible segments or mazes (Table 4). The analysis set is the most valid measure of a child’s language skills compared to the full analysis set, which may suggest the child can produce longer utterances than they can with the inclusion of mazes, etc.

**Table 4:** Micro-structure metric ICC scores.

Metric	ICC	Metric	ICC
Mean length of utterance (words) full transcript	0.911	Present progressives	0.434
Mean length of utterance (morphemes) full transcript	0.832	Questions	0.986
Max length of utterance (words) full transcript	0.986	Subordinate conjunctions	0.772
Max length of utterance (morphemes) full transcript	0.954	Coordinating conjunctions	1.000
Mean length of utterance (words) analysis set	0.904	Regular -s plurals	0.995
Mean length of utterance (morphemes) analysis set	0.924	Irregular plurals	0.664
Max length of utterance (words) analysis set	0.755	`s possessive	0.843
Max length of utterance (morphemes) analysis set	0.793	Articles	0.999
Total utterances	1.000	Regular past tense (−ed)	0.968
Total words	0.998	Irregular past tense	0.984
Keywords	0.987	Third person regular, present tense	0.934
Synonyms of keywords	0.912	Third person irregular, present tense	0.986
Type-token ratio	0.955	Unintelligible words	0.999
Nouns	0.975	Intelligibility	0.998
Pronouns	0.998	Count of mazes	1.000
Verbs	0.987	Comparatives	0.973
Relative pronouns	0.213	Superlatives	0.990
Adverbs	0.884	Contractible copula	0.957
Adjectives	0.949	Uncontractible copula	0.955
Determiners	0.970	Contractible auxiliary	0.904
Particles	0.850	Uncontractible auxiliary	0.870
Prepositions	0.965	Words per minute (*N* = 588)	0.927

ICC reliability = <0.50 = poor; 0.50–0.70 = moderate; 0.70–0.90 = good; >0.90 = excellent (Koo and Li, 2016).

From a total of 44 metrics, the only ICC scores that fell below the ‘good’ level of reliability (<0.70) were relative pronouns, present progressives and irregular plurals.

#### Software testing – Macro-structure analysis module

ICC scores were calculated (Table 5) using subsample D (*N* = 85).

**Table 5:** Macro-structure metrics ICC scores for monolingual replacement samples (*n* = 85).

Macro-structure metric	ICC
Setting	0.850
Initiating event	0.898
Internal response	0.577
Plan	0.841
Attempt	0.616
Consequence	0.729
Character	0.939
Composite macro-structure “story grammar” score*	0.928

ICC reliability = <0.50 = poor; 0.50–0.70 = moderate; 0.70–0.90 = good; >0.90 = excellent (Koo and Li, 2016).

The setting, initiating event, plan and consequence elements had ICC scores categorised as good. Internal response and attempt had ICC scores classified as moderate reliability, and Character had a reliability level of >0.90, which is designated as excellent. When compiled as a total score as a composite of each of the seven macro-structure elements, the total macro-structure story grammar score had an ICC of 0.928, which is classed as excellent reliability (Koo and Li, 2016).

## Discussion

The study demonstrates that it is possible to gather a representative stratified sample of story recall recordings of children aged 4–7 years in the United Kingdom using mobile technology and Citizen Science methods with recordings of sufficient quality for reliable transcription and analysis. Our findings suggest that substantial oversampling is required for such methods to succeed. Approximately 66% of the participants who signed up from the United Kingdom completed all the necessary tasks in the Language Explorer App, including the Story Retell task. Of those 2,340 complete samples, 889 (38%) met the inclusion criteria and were recorded with sufficient quality to be audible. Hence in terms of usable recordings, researchers using these approaches would likely need to oversample by a factor of 2.6. The sample reduces still further when additional exclusion criteria are applied. However, given the low cost of this approach and speed of data acquisition, the method could be of substantial interest to the child language research community. These findings, therefore, address our first two research questions and provide additional information to guide future work of this kind.

Two key caveats must be considered when choosing this method and interpreting our data. First, the partial postcode approach means that the sample is likely to be slightly more advantaged than the United Kingdom population as a whole, as it is likely that more advantaged families within each partial postcode grouping would participate. However, they are likely significantly more representative than many studies in the field of child language, given the speed and low resources needed to recruit families in lower SES postcode areas when compared to the difficulties often experienced by researchers to reach these groups, our data suggest that using a Citizen Science approach using social media, press and paid targeted marketing approaches holds promise for the recruitment of families who are traditionally under-represented in research.

Second, despite instructions not to help children, parents scaffold their child’s narratives to varying degrees to support them in completing the story retelling. Children learn the skill of creating and retelling narratives through social interaction and parents/caregivers’ engagement in narrative co-construction with their child (Wood et al., 1976; Stein and Glenn, 1979), providing scaffolding to support the child to extend and increase the sophistication of their narratives over development. For example, a parent may prompt the child to produce the setting component of the narrative (e.g. ‘where were they going?’) (Stein and Glenn, 1979). This prompt is provided until the child internalises the skill and can use the setting component in their narrative without support. This scaffolding from parents naturally decreases over development in response to the child’s increasing abilities (Bailey and Moughamian, 2007; Bailey et al., 2020). This variability in the implementation of a task is a risk in all Citizen Science data collection (Borda, 2019). A balance must be struck between the benefits of large-scale, low-cost data collection and some variability in task implementation. Our samples have high ecological validity regarding the nature of co-constructed narratives over this developmental period. However, this co-construction makes comparing samples elicited in a clinical context more challenging.

Turning to the research questions regarding the reliability of the automated analysis of micro and macro structure components of the story retell after automatic transcription has been checked and corrected. A set of agreed transcription conventions followed, and the reliability of the micro-structure metrics yielded from the Language Explorer software when compared to SALT software was mostly high. Of the 44 metrics, 33 were excellent, eight were good, one was moderate (irregular plurals), and two were poor (present progressive and relative pronouns). The total macro-structure score ICC, when compared to rating by a trained SLT, was also excellent, indicating it is possible to develop a software platform which can provide reliable macro-structure analyses for a specific story retell. Indeed, the software had good or excellent reliability for all macro-structure elements excepting ‘internal response’ and ‘attempt’ components, suggesting it could provide useful clinical information regarding the overall quality of a child’s narrative macro-structure abilities. We, therefore, recommend further work with clinicians to decide whether some of the least reliable metrics could potentially be dropped entirely from the app’s final reporting output if they are not particularly clinically informative. Also, the final clinical version of the app includes instructions for clinicians regarding the metrics that require manual checking and how to do that.

It must be noted, however, that we have not tested the reliability of these scores regarding the degree to which they represent the child’s broader abilities. There is no explicit agreement in the literature regarding the length of a narrative story retell or spontaneous language samples which provide reliable estimates of a child’s wider abilities (Heilmann et al., 2010; Guo and Eisenberg, 2015; Wilder and Redmond, 2022). In the present study, the narratives that had duration data (*n* = 588) ranged from 35.66 s to 4 min and 19 s, with a mean of 1 min and 48 s (108.26 s). Recently Wilder and Redmond (2022) demonstrated that several language sample metrics (including MLU and number of different words) reach acceptable levels using 3- and 7-min language samples when compared to metrics obtained in 20-min samples. Also, Heilmann and colleagues have demonstrated stable results for productivity and MLU from narrative and other samples of 1–3 min (Heilmann et al., 2010, 2013). Further work to test representativeness compared to a child’s wider language use of the language elicited by narrative retells in general and Language Explorer, in particular, is warranted.

The reliability of the data provided by the Language Explorer App also rests on the accuracy with which the SLT or researcher checks and prepares the language transcript. Following the conventions for utterance segmentation, correctly marking unintelligible utterances, mazes etc., is essential for reliable metrics to be calculated (see Appendix 4). They will also need support to check those few metrics with low reliability identified above. Training materials regarding transcription and analysis checking will therefore need to be included to support clinicians in using the app reliably. Additional work to evaluate this training and other steps in the clinical application of Language Explorer is underway and will be reported elsewhere. Further work would also be needed to assess the clinical use of the story comprehension and repetition subtests.

In terms of acceptability, extremely high numbers of parents reported their children enjoyed the app and found it easy to use. This supports its potential success in its current form for research purposes and is promising in terms of its potential for application in clinical practice. However, Language Explorer will be implemented slightly differently in the clinical context by SLTs, and the acceptability and feasibility of its use in that context will be tested in the clinical evaluation study currently underway.

### Strengths and limitations

The study recruited large numbers of children across a range of socio-economic quintiles and with a wide geographical spread. Furthermore, the majority (66%) of those who signed up completed the tasks within the App. As identified above, due to the use of partial rather than full postcodes, a bias towards more socially advantaged groups than the United Kingdom population is a possible issue (i.e., with the higher SES within each quintile possibly being recruited). However, compared to other research methods and study samples, the Citizen Science approach, linked with a targeted and multi-strategy marketing campaign, appears to be a cost-effective method for reaching subgroups often considered ‘harder to reach’ using more traditional recruitment methods.

Parental scaffolding of narrative retells creates issues comparing these data with retells elicited in more controlled clinical contexts. However, they represent an ecologically valid representation of co-constructed narratives, which form a crucial stage in typical narrative development.

Rigorous training of the RAs and high levels of transcription and analysis reliability among the researchers, together with the large sample (599) to test the micro-structure metrics’ accuracy, provide significant confidence in the study findings. The macro-structure/story grammar analysis module also benefited from the quality and quantity of the data needed to inform the algorithm of the descriptors of each of the seven story grammar elements and the examples for each. A total of 600 samples were used to train the algorithm. Testing with only 85 samples that were not used in training is a limitation. However, the agreement scores for the story grammar elements are promising. In particular, the composite total macro-structure, story grammar score appears robust to measure a complex, discourse-level language feature. It has the potential to be developed to be used to guide assessment in clinical practice, functioning as an indicator of the need or otherwise to examine macro-structure abilities in more detail.

## Conclusion

Language sampling analysis is considered best practice for speech and language therapy assessment of child language (Miller, 1996). The barriers of time and the need for intuitive software to make the process variable for clinicians are established (Klatte et al., 2022) Work presented here has demonstrated the potential of semi-automated transcription and automated linguistic analyses to provide reliable, detailed and informative narrative language analysis for young children and for the use of mobile technologies to collect representative and informative clinical and research data.

A feasibility study of Language Explorer modules is currently underway in clinical settings using the elicitation app and the transcription and analysis tools developed using this dataset. Guidance and training materials were also designed to enable clinical researchers to adhere to the transcription conventions required for reliable automated analyses to be completed in this evaluation phase. This evaluation in a clinical context aims to identify any potential benefits which Language Explorer can bring to clinical practice and what further work would be required to realise them. In this way, we aim to remove critical barriers to using narrative language sample analysis to practice and bring the benefits of more detailed, functional, ecologically valid and sensitive language assessment to the assessment and therapy planning for children with DLD.

## Data availability statement

The datasets presented in this article are not readily available because participants did not provide consent for such access. Requests to access the datasets should be directed to, rbright@therapy-box.co.uk.

## Ethics statement

The studies involving human participants were reviewed and approved by Faculty of Health Sciences Research Ethics Committee University of Bristol. Consent to participate in this study was provided by participants’ parents/carers via the app.

## Author contributions

RB, CM, YW, and EA contributed to the conception and design of the study. EA performed the statistical analysis. RB wrote the first draft of the manuscript. CM and EA wrote sections of the manuscript. All authors contributed to the article and approved the submitted version.

## Funding

This study/project is funded by the NIHR i4i (NIHR200889). The views expressed are those of the author(s) and not necessarily those of the NIHR or the Department of Health and Social Care.

## Conflict of interest

RB is the co-founder of the commercial organisation, Therapy Box, which will commercialise the software described in the article.

The remaining authors declare that the research was conducted in the absence of any commercial or financial relationships that could be construed as a potential conflict of interest.

## Publisher’s note

All claims expressed in this article are solely those of the authors and do not necessarily represent those of their affiliated organizations, or those of the publisher, the editors and the reviewers. Any product that may be evaluated in this article, or claim that may be made by its manufacturer, is not guaranteed or endorsed by the publisher.
