# Supplementary material for: The development of a digital story-retell elicitation and analysis tool through citizen science data collection, software development and machine learning
Source: Front Psychol. 2023 Apr 20;14:989499. doi: 10.3389/fpsyg.2023.989499 (PMC10243469; doi:10.3389/fpsyg.2023.989499)
Supplement: Supplementary file 1 [file Data_Sheet_1.docx]

**Appendix 1**

***Metadata collected***

Gender (male, female)
Birth month and year

Location (the UK, outside the UK)

Language(s) heard at home (first, second)

Speech, language and communication needs

Known other disabilities

***Appendix 2***

***Questions in the parent questionnaire***

1. Did you use the app with a child? (yes, no)
2. Please choose the best answer based on how your child responded to the app: Which of these best describes your relationship to the child? (family, therapist, teacher, other)
3. Please choose the best answer based on how your child responded to the app: Which age group does the child fall in to? (3 years and younger, 4-4.5 years, 5-5.5 years, 5.5-6 years, 6 years, 7 years, 8 years and older)
4. Please choose the best answer based on how your child responded to the app: Does the child have a diagnosed speech or language disability? (no, language disorder, speech sound disorder, fluency disorder, social communication disorder, other)
5. Please choose the best answer based on how your child responded to the app: The child enjoyed using the app (1-10 strongly disagree-strongly agree)
6. Please choose the best answer based on how your child responded to the app: The child found the tasks hard (1-10 strongly disagree-strongly agree)
7. Please choose the best answer based on how your child responded to the app: Which task was the child’s favourite? (story retell, quiz, repeating sentences)
8. Please choose the best answer based on how your child responded to the app: The child understood the tasks without any instruction from an adult? (1-10 strongly disagree – strongly agree)
9. Please choose the best answer based on how your child responded to the app: The child liked to play the game (1-10, strongly disagree – strongly agree)
10. Select the best response which matches your response to the app: I liked the app (1-10, strongly disagree – strongly agree)
11. Select the best response which matches your response to the app: I understand the purpose of the app (1-10, strongly disagree – strongly agree)
12. Select the best response which matches your response to the app: I would recommend this app to others (1-10, strongly disagree – strongly agree)
13. Select the best response which matches your response to the app: The app was easy to use (1-10, strongly disagree – strongly agree)

**Appendix 3**

***Process for determining SES quintile***

|  | **Input** | **Mapping** | **Aggregate** | **Rank** | **Stratify** |
| --- | --- | --- | --- | --- | --- |
|  | 1 | 2 | 3 | 4 | 5 |
| England | Partial Postcode | GeoConvertor  () 1. Match one geography onto another 2. Source geography = postcode district 3. Target Geography = LSOA Generate table | 1. Go to English IMD and download file 1 ([https://www.gov.uk/government/statistics/english-indices-of-deprivation-2019](https://eur03.safelinks.protection.outlook.com/?url=https%3A%2F%2Fwww.gov.uk%2Fgovernment%2Fstatistics%2Fenglish-indices-of-deprivation-2019&data=04%7C01%7Ccristina.mckean%40newcastle.ac.uk%7C8716aeac82a741084f9a08d9319e29e8%7C9c5012c9b61644c2a91766814fbe3e87%7C1%7C0%7C637595377642929089%7CUnknown%7CTWFpbGZsb3d8eyJWIjoiMC4wLjAwMDAiLCJQIjoiV2luMzIiLCJBTiI6Ik1haWwiLCJXVCI6Mn0%3D%7C1000&sdata=9w8z%2F3m35g9YI%2BV4YuGYI4nTFVrbQtJCX%2BlCPx4TE2Y%3D&reserved=0)) 2. Table from file 1 shows IMD rank for each LSOA. 3. Use both this table and the table from C4 to find an average rank of all of the  LSOA in a partial postcode (sum of IMD rank for LSOA in a partial postcode/ n of  LSOA in a partial postcode) | Rank the average rank of partial postcode | Split the average rank of partial postcodes into quintiles |
| Scotland | Partial Postcode | GeoConvertor Use same table as C4 | 1. Go to Scottish IMD (Scottish Index of Multiple Deprivation 2020v2 - ranks) 2. Table from file 1 shows IMD rank for each LSOA (Called a data zone in database). 3. Use both this table and the table from C4 to find an average rank of all of the  LSOA in a partial postcode (sum of IMD rank for LSOA in a partial postcode/ n of  LSOA in a partial postcode) | Rank the average rank of partial postcode | Split the average rank of partial postcodes into quintiles |
| Wales | Partial Postcode | GeoConvertor Use same table as C4 | 1. Go to Welsh IMD and download WMD 2019 Index and domain score by small area 2. Table from file 1 shows IMD rank for each LSOA (Called a data zone in database). 3. Use both this table and the table from C4 to find an average rank of all of the  LSOA in a partial postcode (sum of IMD rank for LSOA in a partial postcode/ n of  LSOA in a partial postcode) | Rank the average rank of partial postcode | Split the average rank of partial postcodes into quintiles |
| Northern Ireland | Partial Postcode | GeoConvertor Use same table as C4 | 1. Go to NI IMD and download SOA level results 2. Table from file 1 shows IMD rank for each LSOA (Called a soa in database). 3. Use both this table and the table from C4 to find an average rank of all of the  LSOA in a partial postcode (sum of IMD rank for LSOA in a partial postcode/ n of  LSOA in a partial postcode) | Rank the average rank of partial postcode | Split the average rank of partial postcodes into quintiles |

**Appendix 4**

***Transcription conventions***

1. Transcribe utterances into C-units. A C-unit includes one main clause with all subordinate clauses attached to it. It cannot be further divided without the disappearance of its essential meaning.

- Where there are coordinating conjunctions, which link two main clauses, they should be separated/segmented into two utterances (or two C-units) that can each stand alone e.g., Suzy had a nap (one C-unit/utterance). And Tom climbed the tree (one C-unit/utterance). Both can stand on their own and still make sense.
- For subordinating conjunctions, you would transcribe the main clause and subordinating clause as one C-unit e.g., Suzy had a nap *while* Tom climbed the tree. (If you segmented them into two, the second part wouldn't make sense on its own).
- Exceptions to this would be if the child was interrupted or if they were answering a question, then the subordinating clause could be transcribed as one c-unit.

1. Do not transcribe pauses.
2. Punctuation to use:

- First line of utterance is capitalised and capitals for names.
- All utterances end in a full stop/question mark/exclamation mark as appropriate.
- Do not use commas or speech marks (for indirect speech).

1. Identifiable information e.g., places or names that are not Tom/Suzy/Jack, need to redacted with - - name- -.
2. Mazes are classed as repetitions, false starts, fillers such as ‘erm’ etc. These need to be put in brackets. For mazes, mark the first set of repetitions rather than the second (as it's likely the first set has been self-corrected/re-modelled for the second set which carries the meaning in the utterance) e.g. (She dug) she dug for treasure.
3. Unintelligible segments are marked as XXX. Do not put XXX within mazes/brackets.
4. Use the transcription conventions for speech sound errors and other mispronounced words e.g., if the child says “gonna”, you type: /gonna/[going to], if the child says “cwossed”, you type /cwossed/[crossed]

**Appendix 5**
*Agreement Levels between RAs and Language Explorer after Piloting with subsample A*

| **Metric** | **Initial phase 85% agreement** | **Metric** | **Final phase 85% agreement** |
| --- | --- | --- | --- |
| Mean length of utterance (words) full transcript | Achieved | Mean length of utterance (words) full transcript | Achieved |
| Mean length of utterance (morphemes) full transcript | Achieved | Mean length of utterance (morphemes) full transcript | Not achieved |
| Max length of utterance (words) full transcript | Achieved | Max length of utterance (words) full transcript | Achieved |
| Max length of utterance (morphemes) full transcript | Not achieved | Max length of utterance (morphemes) full transcript | Achieved |
| Mean length of utterance (words) analysis set | Achieved | Mean length of utterance (words) analysis set | Achieved |
| Mean length of utterance (morphemes) analysis set | Achieved | Mean length of utterance (morphemes) analysis set | Achieved |
| Max length of utterance (words) analysis set | Achieved | Max length of utterance (words) analysis set | Achieved |
| Max length of utterance (morphemes) analysis set | Not achieved | Max length of utterance (morphemes) analysis set | Achieved |
| Total utterances | Achieved | Total utterances | Achieved |
| Total words | Achieved | Total words | Achieved |
| Keywords | Achieved | Keywords | Achieved |
| Type-token ratio | Achieved | Type-token ratio | Achieved |
| Synonyms of keywords | Not achieved | Synonyms of keywords | Achieved |
| Nouns | Achieved | Nouns | Achieved |
| Pronouns | Achieved | Pronouns | Achieved |
| Verbs | Achieved | Verbs | Achieved |
| Determiners | Achieved | Determiners | Achieved |
| Questions | Achieved | Questions | Achieved |
| Coordinating conjunctions | Achieved | Coordinating conjunctions | Achieved |
| Prepositions | Not achieved | Prepositions | Achieved |
| Regular -s plurals | Achieved | Regular -s plurals | Achieved |
| Irregular plurals | Achieved | Irregular plurals | Achieved |
| `s possessive | Achieved | `s possessive | Achieved |
| Articles | Achieved | Articles | Achieved |
| Regular past tense (-ed) | Achieved | Regular past tense (-ed) | Achieved |
| Irregular past tense | Not achieved | Irregular past tense | Achieved |
| Third person regular, present tense | Achieved | Third person regular, present tense | Achieved |
| Third person irregular, present tense | Not achieved | Third person irregular, present tense | Achieved |
| Unintelligible words | Achieved | Unintelligible words | Achieved |
| Intelligibility | Achieved | Intelligibility | Achieved |
| Count of mazes | Not achieved | Count of mazes | Achieved |
| Words per minute | Achieved | Words per minute | Achieved |
| Comparatives | Achieved | Comparatives | Achieved |
| Superlatives | Achieved | Superlatives | Achieved |
| Contractible copula | Achieved | Contractible copula | Achieved |
| Uncontractible copula | Not achieved | Uncontractible copula | Achieved |
| Contractible Auxiliary | Not achieved | Contractible Auxiliary | Achieved |
| Uncontractible Auxiliary | Not achieved | Uncontractible Auxiliary | Not achieved |
| Adverbs | Not achieved | Adverbs | Not achieved |
| Adjectives | Not achieved | Adjectives | Not achieved |
| Present progressives | Not achieved | Present progressives | Not achieved |
| Subordinate conjunctions | Not achieved | Subordinate conjunctions | Not achieved |
| Particles | Not achieved | Particles | Not achieved |
| Relative pronouns | Not achieved | Relative pronouns | Not achieved |

**Appendix 6**

***Levels of reliability between RAs for transcription and language analysis of micro- and macro-structure features***

| Orthographic transcription subsample A | Average agreement |
| --- | --- |
| RA1 | 98.94% |
| RA2 | 97.05% |
| RA3 | 97.77% |

| Orthographic transcription of subsample B | Average agreement |
| --- | --- |
| RA1 | 94.38% |
| RA2 | 94.23% |
| RA3 | 93.19% |

| Orthographic transcription of subsample C | Average agreement |
| --- | --- |
| RA1 | 94.22% |
| RA2 | 94.16% |
| RA3 | 93.43% |

| Language analysis subsample A | Average agreement |
| --- | --- |
| RA1 | 97.58% |
| RA2 | 96.02% |
| RA3 | 96.11% |

| Language analysis of subsample C | Average agreement |
| --- | --- |
| RA1 | 98.91% |
| RA2 | 98.61% |
| RA3 | 98.54% |

| Language analysis of subsample B | Average agreement |
| --- | --- |
| RA1 | 98.89% |
| RA2 | 98.53% |
| RA3 | 98.45% |
| Macro-structure subsample A attempt 1 | Macro-structure components (average ICC) |
| RA1 | 0.455 |
| RA2 | 0.562 |
| RA3 | 0.587 |

| Macro-structure subsample A attempt 2 | Macro-structure components (average ICC) | Macro-structure total score (average ICC) |
| --- | --- | --- |
| RA1 | 0.885 | 0.938 |
| RA3 | 0.940 | 0.938 |

| Macro-structure subsample B | Macro-structure components (average ICC) | Macro-structure total score (average ICC) |
| --- | --- | --- |
| RA1 | 0.907 | 0.956 |
| RA3 | 0.869 | 0.925 |

| Macro-structure subsample C | Macro-structure components (average ICC) | Macro-structure total score (average ICC) |
| --- | --- | --- |
| RA1 | 0.907 | 0.956 |
| RA3 | 0.872 | 0.934 |

**Appendix 7`**

***Example narratives for each age banding***

**4;0-4;5 years**

T(So what's hap) so you want to start telling the story then?

CNo.

TWhat's happening now?

C(Um) they're actually going on a boat.

TThey're on a boat.

CThey jumped off.

C(Um) can I not do the rest?

CCan you do the rest?

TOh no it's your turn.

TYou were really good at this.

T--name-- what's happening next?

CNow they /gonna/[going to] walk over that bridge aren't they.

CThen.

TWhat are they doing now?

CCan I press it?

TYeah in a minute.

TWhat are they doing now?

CThey're actually going across the (wo wib wob wob) wobbly bridge.

TOK.

CCan I press it?

TYep.

TWhat's they doing now?

CNow they're /gonna/[going to] climb the tree.

TOK.

TGo on then.

CThen they found the rock.

TGo on then.

CDo you know what kind of thing is that?

CIt's a tortoise.

CI like all kind of animals.

CJust the ones what I do know.

CI don't know the ones what I don't know.

TOK.

TAre you going to carry on telling the story?

TWhat next?

CSad.

CSee.

TThey were feeling sad.

TWhat happened next?

CThe map is stolen remember.

TYeah.

CBut then.

TGo on then.

C(Then) then they are trying to get after them.

CBut he was angry.

THe was angry.

CYeah.

TI can imagine.

CWhat?

TPress that one.

TThat's it.

CXXX.

CThen.

TWhere did they get to?

C(They actually get to don't they) they get to another rock.

TAha.

CAnd it's the same as the tortoise.

TIt looks the same as the tortoise.

CI think it's a mummy tortoise.

TGo on then.

C(Then they found) they digging (the food) the x marks the spot aren't they.

TYeah.

CThen they found the treasure.

CAnd they left the bone there didn't they.

CBut where's his bone?

T/Dunno/[don't know].

CIs that his bone?

COr is that the map?

TOh i don't know.

TIt could be the map.

TAre you finished?

CYeah.

**4;6-4:11 years**

CXXX sunny day.

TYeah.

TNext.

TGo on then.

C(He got back off the boat quickly) he got back (on the) off the (cl) boat quickly.

C(And then he f) and then he opened his map.

CAnd then (he saw) he went across the (wobbly wobbly thing) wobbly bridge.

CAnd he was stepping over (it) bridge carefully.

C(And then he) and then he climbed up (a big) the tallest tree while his dog was snoring.

CAnd he pointed to the rock.

C(And then) and then it wasn't there.

CAnd then the dog /digged/[dug] fast as he could.

CAnd then it was a turtle.

C(And then a parrot) and then his helping XXX XXX.

COne of the cheeky little parrots trying to (get) get (the thing) the map.

CAnd then the parrot took it.

CAnd the doggy (and) trying to find it.

C(And then he) and then they finally found the one.

CAnd doggy /digged/[dug].

C(He) he finally found the treasure.

**5;0-5;5 years**

COne sunny day (ah).

TThat's OK.

TJust say what you remember.

CThere was a boy called Tom and a dog called Suzy.

C(They were) Oh can you get on back on the other one?

TNo you can't get back I don't think.

TIt's OK.

TSay what you want to say.

CThey sailed on the sea to an island.

CAnd when they got there they started to look on the map.

CThey looked for the treasure where they could see.

C(Tom) Suzy took (asleep) a nap while Tom climbed the biggest tree.

CTom spotted (um) the rock.

CSuzy started to sniff.

C(Oh no it isn't) It's an animal.

CIs it (a tur) a tortoise said Tom.

CIt came really disappointed.

CIt went even disappointed parrots started to grab their treasure map.

C(They ran as fast as they could) They ran as fast as they could.

CCatch that map!

CSuzy started to yell.

CThey found the treasure.

C(When they) Suzy started to dig.

CThey found the most /goldish/[golden] treasure.

CWhich one do I press?

TThat's it.

TThat one there.

TWell done!

**6;0-6;11 years**

COne sunny day Tom and Suzy went on a adventure to find some lost treasure.

CWhen they reached the island they quickly jumped off the boat.

CTom pointed at the map where the treasure was hidden.

CHe pointed at a rock.

CThey crossed the wobbly bridge.

CTom decided to climb the highest tree to spot the treasure.

CBut Suzy napped on the ground.

CTom pointed to the rock that was on the map.

CThe treasure was buried under it or was it?

CThen Suzy started sniffing as quickly as she could.

CIt moved.

CIs that a tortoise said Tom?

CThen that's when they realised finding the treasure (wasn't) would be quite hard and difficult and very very very hard indeed.

CThey were very very sad.

CAnd two cheeky parrots flew off with their map which made it even more (hard hard hard) hard.

CTom and Suzy ran after them until they got to the rock which the parrots had landed on.

CSuzy started digging dig dig dig dig dig dig dig as fast as her little paws could go.

CThey were so happy when they finally (uncar) uncovered the treasure.

**7;0-7;11 years**

COne sunny day there was a man and a dog sailing (for a treasure) to try and find the best treasure.

CWhen they got to the island they were excited and hopped off.

CAfter that (they) they looked around on the map and found a bridge to cross on.

CThey crossed the /bidge/[bridge] (carefully as carefully as th) as carefully as they could and got off.

CAfter a while (--name-- got) the dog --name-- got a bit tired and had a rest.

CWhile (--name-- climbed the) Tom climbed the highest tree (to find) to try and find the rock.

C(He found the rock) and he found the rock up the tree.

CThey went to it.

C(And) and it started to move.

CAnd it was a tortoise.

C--Name-- got so sad and --name-- did as well.

CThen suddenly some parrots swooped over (and took the) and took the map.

C(And) and they were furious and ran after the parrots.

CBut then --name-- managed to catch up and brought them to the rock (that looked like) that looked like the one on the map.

CAnd then (they started) --name-- started digging.

C(And --name--) and Tom got excited.

Finally, they found the beautiful glistening treasure.
